# Sharing Solidarity Experiences to Overcome COVID-19

**DOI:** 10.5334/aogh.3035

**Published:** 2020-09-03

**Authors:** Morteza Arab-Zozani, Soheil Hassanipour

**Affiliations:** 1 Social Determinants of Health Research Center, Birjand University of Medical sciences, Birjand, IR; 2 Gastrointestinal and Liver Diseases Research Center, Guilan University of Medical Sciences, Rasht, IR

## Abstract

Solidarity in the general sense means unity or agreement of feeling or action, especially among individuals with a common interest; or mutual support within a group. There are different ways of standing in solidarity in different kinds of literatures. One of the most important ways is to advocate. Advocacy is a win-win strategy and a process of supporting and enabling people to express their views and concerns. In the end, I think sharing different types of solidarity can be one of the drivers that stimulate the solidarity itself, and I call on everyone to contribute to this sharing. I hope that this solidarity, which began in the world with the beginning of COVID-19, will not end with its end and will last forever because our world needs coexistence. This may be the only gift to the world from COVID-19.

**Dear editor,** 

A few days after the invasion of COVID-19 far from the borders of China and its signs appearing around the world, Dr. Tedrus Adhanom, director general of the World Health Organization (WHO), spoke of a word called solidarity [1,2]. Solidarity in the general sense means unity or agreement of feeling or action, especially among individuals with a common interest; or mutual support within a group [3].

Throughout history, solidarity has often been a tool for the implementation of equality and the reduction of social injustice. One example is the labor movement of the same name in Poland in 1981, which began to oppose communist regimes [4]. There are also other cases of solidarity on issues such as gender-based violence or human trafficking by the Anglican Alliance [5].

Solidarity is an individual or group value aimed at the sustainable development of peoples and communities. It works in such a way that it can provide unity between different individuals and communities to achieve better well-being, and better well-being than health for all, which has been an eternal slogan for the WHO [6].

According to Emile Durkheim, a well-known sociologist, there are two types of solidarity: mechanical and organic. Organic solidarity occurs when a society is maintained by the division of labor. On the other hand, mechanical solidarity occurs when a society is maintained by the similarities of its people [7]. Regardless of the type of solidarity that underlies the director of the WHO, this has played a vital role in the fight against COVID-19. In this article, we want to ask all the people of the world to give examples of the solidarity they have shown in their community or the international community because we believe that the spread of these unique experiences can spread happiness and love in the world. To that end, we have outlined simple ways to stand in solidarity.

There are different ways of standing in solidarity in different kinds of literatures. One of the most important ways is to advocate. Advocacy is a win-win strategy and a process of supporting and enabling people to express their views and concerns. It is also an action by a person or group aimed to influence decisions within different institutions. Another type of solidarity is to give. This can be to donate things that we don’t use but might be needed by someone else. It is believed that the most important thing that can be given to others is a listening ear. The third type of solidarity is to learn. Learning refers to the acquisition of knowledge about things we do not know. This learning can be about all things related to the disease, such as symptoms, ways of transmission, methods of prevention and treatment. Education also is a powerful weapon used for learning. Another type of solidarity is to speak up. Talking about inequality and injustice plays a vital role in today’s society and can be a powerful catalyst for change and to establish justice. Another simple way to promote solidarity is to be kind. Being kind to people or communities who are struggling with a problem can increase their ability to overcome it [8,9,10]. Studies have also shown that kindness to people with problems can strengthen the immune system and better respond to disease. Most of the aid currently available worldwide can be found in one of these five types. Things like giving heart to sick patients by talking to them or listening to them or sending encouraging messages, playing a melody, singing a song, financial aid, humanitarian aid such as giving meals or health care packages, and even smiling at patients and healthcare staff can be different examples of this solidarity.

In the end, We think sharing different types of solidarity can be one of the drivers that stimulate solidarity itself, and I call on everyone to contribute to this sharing. I hope that this solidarity, which began in the world with the beginning of COVID-19, will not end with its end and will last forever because our world needs coexistence. This may be the only gift to the world from COVID-19.
